# Supplementary figures and images for: Antero-Posterior vs. Lateral Vestibular Input Processing in Human Visual Cortex
Source: Front Integr Neurosci. 2020 Aug 10;14:43. doi: 10.3389/fnint.2020.00043 (PMC7430162; doi:10.3389/fnint.2020.00043)

Supplementary Figure1:

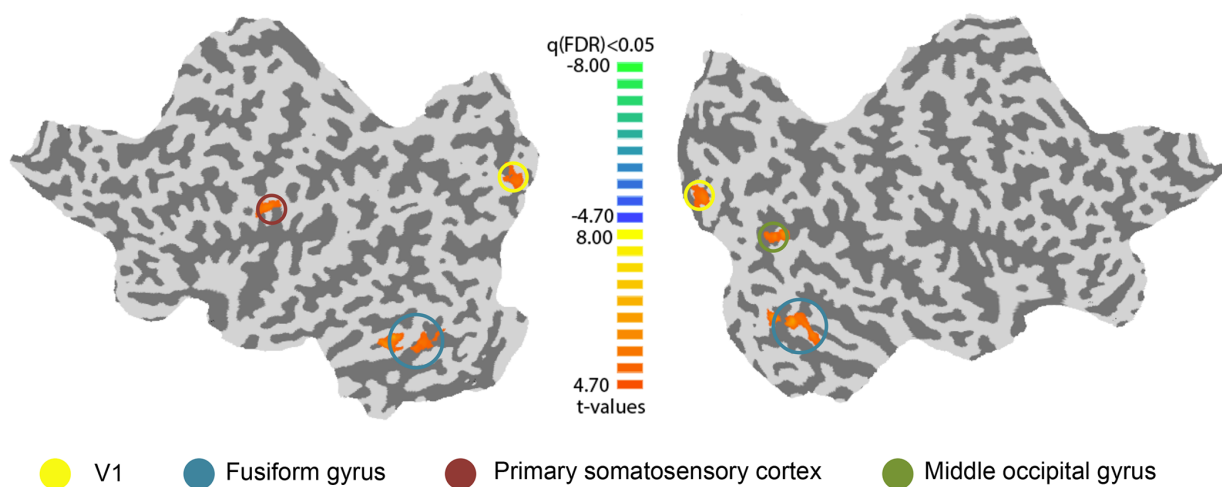

Supplementary Figure2:

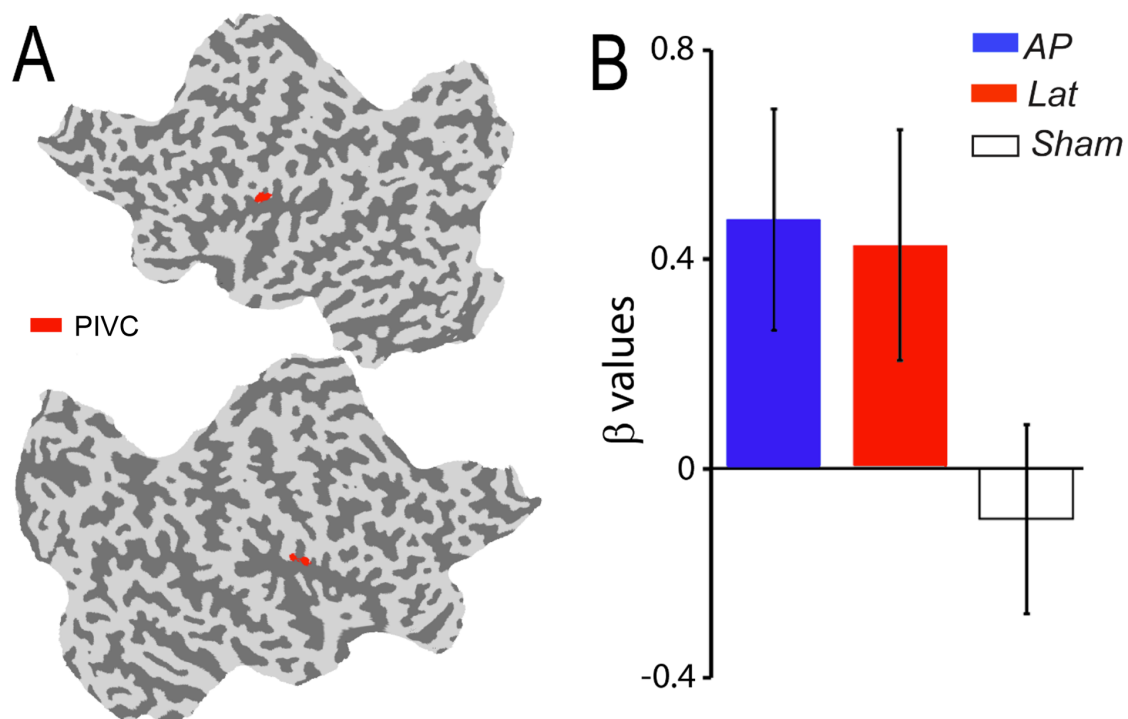

Supplement: FIGURE S1 — Activations during sham stimulation: group analysis (n = 13) of cortical activations during sham stimulation. Bilateral activation was found in V1 (Talairach space, −3, −75, 13 and 5, −73, 11 for left and right hemisphere, respectively) and fusiform gyrus (Talairach space, −27, −63, −7 and 26, −60, −9 for left and right hemisphere, respectively). Also, significant activation was found in the left primary somatosensory cortex (Talairach space, −56, −15, 13) and right middle occipital gyrus (Talairach space, 29, −77, 18). [file Data_Sheet_1.PDF]
